# The Importance of MRI in Accurately Diagnosing Cavernous Angioma/Cavernoma: A Case Report

**DOI:** 10.51894/001c.144461

**Published:** 2025-09-30

**Authors:** Jay Garlapati

**Affiliations:** 1 Department of Internal Medicine Detroit Medical Center-Sinai Grace Hospital

## 81

### INTRODUCTION

Cavernous angiomas, also known as cavernomas, are vascular malformations characterized by clusters of dilated blood vessels that can lead to significant neurological complications, including seizures, hemorrhages, and focal neurological deficits. We present a case of an elderly male who was initially thought to have an intraparenchymal hemorrhagic stroke but was later found to have a cavernoma instead. This case highlights the critical role of MRI in accurately diagnosing and managing cavernous angiomas, particularly in patients with confounding clinical and radiological findings.

### CASE DESCRIPTION

The patient is an 81-year-old male with a history of colon cancer, COPD, hypertension, and dementia who presented to the Emergency Department with altered mental status. The patient was found by his wife after attempting to use the bathroom but was unable to return to his room and was found seated in the hallway. Vitals were significant for severely elevated blood pressure and low oxygen saturation. A CT head revealed an acute 16 x 13 mm intraparenchymal hemorrhage in the left posterior basal ganglia and a CTA showed a hyperdense lesion in the left deep temporal lobe with small caliber tortuous vascularity suspicious for a mass or vascular malformation. An MRI performed a few days later revealed a 13 x 10 x 12 mm cavernous angioma/cavernoma in the left temporal lobe as well as old hemorrhages in the midbrain and bilateral temporal lobes and old infarctions in the right basal ganglia/anterior limb of the midbrain and posterior occipital lobe.

### DISCUSSION

Cavernous angiomas are abnormal clusters of dilated, thin-walled blood vessels, devoid of intervening brain tissue. Cavernomas, whether symptomatic or incidentally discovered, can be accurately diagnosed via MRI, which offers nearly 100% sensitivity. On T2 weighted MRI, cavernomas typically demonstrate a characteristic mixed signal “popcorn” core with a hypointense rim. We recommend performing a brain MRI as soon as possible for patients with a suspected intraparenchymal hemorrhage to enable prompt and accurate diagnosis. This approach ensures timely initiation of the appropriate treatment—whether surgical excision or targeted radiotherapy—avoiding delays and minimizing the risk of adverse outcomes due to incorrect treatment protocols or disease progression.

